# The STRIPAK component SipC is involved in morphology and cell-fate determination in the nematode-trapping fungus *Duddingtonia flagrans*

**DOI:** 10.1093/genetics/iyab153

**Published:** 2021-09-25

**Authors:** Valentin Wernet, Jan Wäckerle, Reinhard Fischer

**Affiliations:** Department of Microbiology, Institute for Applied Biosciences, Karlsruhe Institute of Technology (KIT)—South Campus, D-76131 Karlsruhe, Germany; Department of Microbiology, Institute for Applied Biosciences, Karlsruhe Institute of Technology (KIT)—South Campus, D-76131 Karlsruhe, Germany; Department of Microbiology, Institute for Applied Biosciences, Karlsruhe Institute of Technology (KIT)—South Campus, D-76131 Karlsruhe, Germany

**Keywords:** striatin-interacting phosphatase and kinase (STRIPAK) complex, nematode-trapping fungi, Duddingtonia flagrans, fungal development, trap formation, septation

## Abstract

The striatin-interacting phosphatase and kinase (STRIPAK) complex is a highly conserved eukaryotic signaling hub involved in the regulation of many cellular processes. In filamentous fungi, STRIPAK controls multicellular development, hyphal fusion, septation, and pathogenicity. In this study, we analyzed the role of the STRIPAK complex in the nematode-trapping fungus *Duddingtonia flagrans* which forms three-dimensional, adhesive trapping networks to capture *Caenorhabditis elegans*. Trap networks consist of several hyphal loops which are morphologically and functionally different from vegetative hyphae. We show that lack of the STRIPAK component SipC (STRIP1/2/HAM-2/PRO22) results in incomplete loop formation and column-like trap structures with elongated compartments. The misshapen or incomplete traps lost their trap identity and continued growth as vegetative hyphae. The same effect was observed in the presence of the actin cytoskeleton drug cytochalasin A. These results could suggest a link between actin and STRIPAK complex functions.

## Introduction

Polar growth, polarity establishment, and maintenance of polarity of cells are common themes in biology and largely depend on cytoskeletal elements and functions. A *bona fide* example of polar growth is filamentous fungi with their extremely elongated hyphae (Riquelme *et al.* 2018). Most fungal species grow apically by continuous cell wall and cell membrane extension. Secretion vesicles containing, *e.g.*, cell wall biosynthesis enzymes are transported along microtubules (MTs) toward the tip, accumulate transiently in a so-called *vesicle supply center* or *Spitzenkörper* from where they are transported toward the apical membrane. After fusion with the membrane, they deliver their protein content (Riquelme *et al.* 2007). For the last travel distance, the actin cytoskeleton is required (Bergs *et al.* 2016; Schultzhaus *et al.* 2016). The MT and the actin cytoskeletons are coordinated through the action of so-called *cell-end marker proteins* at the cortex (Mata and Nurse 1997; Takeshita *et al.* 2013, 2014, 2017). Any variation from the tube-like morphology of hyphae is probably preceded and accompanied by cytoskeletal rearrangements.

One intriguing fungal hyphal morphology is the ring-like structure of traps of nematode-trapping fungi (NTF) (Jiang *et al.* 2017). These fungi can switch from a saprotrophic to a predatory lifestyle under low-nutrient conditions and in the presence of nematodes. More than 200 NTF have been identified and many are found in the Orbiliaceae (Ascomycota). A wide range of morphologically different traps are produced by different species, ranging from adhesive knobs and columns, adhesive trap rings and networks, and constricting and non-constricting rings (Su *et al.* 2017). One of the best-studied NTF at the molecular level is *Arthrobotrys oligospora*, where also the signal exchange between fungus and nematodes and the control of trap formation were studied (Niu and Zhang 2011; Hsueh *et al.* 2013, 2017; Yu *et al.* 2021)*.* In the case of *Duddingtonia flagrans*, a close relative of *A. oligospora*, ring-like, adhesive traps are formed, in which *Caenorhabditis elegans* or other nematodes are captured (Youssar *et al.* 2019). The ring-like morphology of the traps is rather unique and requires a redirection of the polarity axis, cell–cell recognition, and hyphal fusion. This morphological differentiation goes along with physiological differentiation, the production of adhesives, and the preparation for the penetration of caught nematodes. Another fascinating aspect is the determination of the size of the traps. They should have a defined diameter to effectively catch nematodes. If they are too large, nematodes could slip through, and if the diameter is too small, nematodes will not be able to enter the traps. Size determination probably also requires a functional cytoskeleton. How these fascinating morphological changes are initiated and which signaling modules are required are largely unknown.

Although some trapping-deficient mutants have been identified in *A.* *oligospora*, the molecular basis and cell biology of trap morphogenesis are poorly understood (Kuo *et al.* 2020; Chen *et al.* 2021). Recently, a random mutagenesis analysis identified 15 mutants with deficiencies in trap morphogenesis. However, there are still difficulties in gene mapping because of heterokaryotic transformants and the lack of genetic crosses of NTF (Huang *et al.* 2021). In *D.* *flagrans*, it was shown that a cell dialog between the tip trap cell and the basal cell is required for ring closure (Youssar *et al.* 2019).

The early stages of trap formation in *D. flagrans* resemble the spiral growth of ascogonia or crozier formation of ascomycetes during sexual development (Read and Beckett 1996). An ascogenous hypha elongates and bends over to form a hook-shaped structure. In *Sordaria macrospora*, a genetic screen has identified several “*pro*” mutants with defects in sexual development, and some PRO proteins are components of the *striatin-interacting phosphatase and kinase* (STRIPAK) complex (Stein *et al.* 2020; Teichert *et al.* 2020). The STRIPAK complex is a highly conserved eukaryotic signaling module responsible for the regulation of numerous cellular and developmental processes. It was initially identified in mammals, where it was linked to human diseases like Alzheimer’s, diabetes, and cardiac diseases (Goudreault *et al.* 2009; Hwang and Pallas 2014). Recently, the structure of the human STRIPAK core was solved by cryo-EM (Jeong *et al.* 2021). The serine–threonine protein phosphatase 2 (PP2A) core enzyme consists of a scaffold subunit A (PP2A), the catalytic subunit C (PP2AC), and a regulatory subunit of the B′′′ (striatin) family. In addition, multiple subunits are associated with the core subunits of the phosphatase, including the striatin-interacting protein 1 or 2 (STRIP1 or STRIP2), the monopolar spindle one-binder (Mob) protein Mob3, cerebral cavernous malformation 3, the sarcolemmal membrane-associated protein (SLMAP), and the coiled-coil protein suppressor of IκB kinase-ε. Furthermore, various germinal center kinases (GCKs) are associated with the STRIPAK complex for enhanced regulation.

The STRIPAK complex is involved in the regulation of key cellular processes including signaling, cytoskeletal organization, cell cycle control, cell migration, cell polarity, apoptosis, Golgi assembly, cell morphology, and vesicular trafficking (Hwang and Pallas 2014; Kück *et al.* 2019). In *Drosophila melanogaster*, it was shown that the STRIPAK complex is a negative regulator of the Hippo signaling pathway through its phosphatase activity (Ribeiro *et al.* 2010). RNAi screening identified the STRIP1/2 orthologs FAM40A and FAM40B to affect cell shape in Drosophila S2 cells (Rohn *et al.* 2011). In an RNAi screening in PC3 prostate cancer cells, FAM40A-depleted cells were flattened with high levels of cortical F-actin compared to the control, whereas FAM40B-depleted cells were elongated with long thin protrusions (Bai *et al.* 2011). In *Saccharomyces cerevisiae*, the STRIPAK homolog Factor arrest protein complex (Far complex) is involved in pheromone-induced cell cycle arrest and affects signaling through the target of rapamycin complex 2 (Kemp and Sprague 2003; Pracheil *et al.* 2012). In *Schizosaccharomyces pombe*, the STRIPAK homologous Septation initiation network (SIN) Inhibitory PP2A complex (SIP) negatively regulates SIN and is important for coordinating mitosis and cytokinesis (Singh *et al.* 2011; Simanis 2015).

Functional analyses in filamentous fungi revealed that different components are required for cell–cell fusion, sexual fruiting body development, septation, secondary metabolite production, symbiotic interactions, and pathogenicity (Xiang *et al.* 2002; Pöggeler and Kück 2004; Green *et al.* 2016; Zhang *et al.* 2018; Elramli *et al.* 2019). But only recent proteomic and phosphoproteomic studies in *S. macrospora* revealed an extended signaling network that is modulated by the STRIPAK complex (Märker *et al.* 2020).

Here, we studied the STRIPAK complex in the NTF *D. flagrans*, investigated the role of the STRIPAK complex component SipC/STRIP1/2 during septation and the unique hyphal ring formation during trap morphogenesis, and found that SipC is involved in cell-fate determination.

## Material and methods

### Strains and culture conditions

*D. flagrans* strains were cultivated at 28°C on potato dextrose agar (PDA; Carl Roth). All fungal strains used in the study are listed in Table 1.

**Table 1:** *D. flagrans* strains used in this study

Strain		Reference
WT	CBS 349.94	CBS-KNAW
sVW03	*sipC::hph (ΔsipC); trpC(p)::hph::trpC(t)*	This study
sVW05	*ΔsipC; sipC(p)::sipC::sipC(t); gpdA(p)::neo::trpC(t)*	This study
sVW10	*h2b(p)::h2b::GFP::h2b(t); gpdA(p)::neo::trpC(t)*	This study
sVW15	*ΔsipC*; *tubA(p)::mCherry::tubA::tubA(t); gpdA(p)::neo::trpC(t)*	This study
sVW16	*ΔsipC*; *tubA(p)::lifeact::GFP::gluC(t); gpdA(p)::neo::trpC(t)*	This study
sVW18	*tubA(p)::mCherry::tubA::tubA(t); trpC(p)::hph::trpC(t)*	This study
sVW22	*ΔsipC*; *h2b(p)::h2b::GFP::h2b(t); gpdA(p)::neo::trpC(t)*	This study
sVW24	sVW10 + *sipC(p)::h2b::mCherry::h2b(t); trpC(p)::hph::trpC(t)*	This study
sVWZ	*tubA(p)::lifeact::GFP::gluC(t); trpC(p)::hph::trpC(t)*	This study
sVW X	*p12(p)::p12::mCherry::gluC(t); trpC(p)::hph::trpC(t)*	This study
sVW Y	*ΔsipC; p12(p)::p12::mCherry::gluC(t); gpdA(p)::neo::trpC(t)*	This study

**Table 2:** Plasmids used in this study

Name	Description	Reference
pVW28	*sipC*::*hph* (*ΔsipC*)	This study
pJW04	*h2b(p)::h2b::GFP::h2b(t); gpdA(p)::neo::trpC(t)*	This study
pVW37	*sipC(p)::sipC::sipC(t); trpC(p)::neo::trpC(t)*	This study
pVW41	*lifeact*::*GFP*	This study
pVW42	*tubA(p)::lifeact::GFP; trpC(p)::hph::trpC(t)*	This study
pVW56	*p12(p)::h2b::mCherry::gluC(t); trpC(p)::hph::trpC(t)*	This study
pVW57	*tubA(p)::mCherry::tubA::tubA(t); trpC(p)::hph::trpC(t)*	This study
pVW70	*sipC(p)::h2b::mCherry::h2b(t); trpC(p)::hph::trpC(t)*	This study
pVW77	*tubA(p)::mCherry::tubA::tubA(t); gpdA(p)::neo::trpC(t)*	This study
pVW78	tubA(p)::lifeact::GFP; gpdA(p)::neo::trpC(t)	This study
pVW114	p12(p)::p12::mCherry::gluC(t); trpC(p)::hph::trpC(t)	This study
pVW115	p12(p)::p12::mCherry::gluC(t); gpdA(p)::neo::trpC(t)	This study
pIH02	*pplasmid backbone containing trpC(p)::hph::trpC(t)*	Youssar *et al.* (2019)
pJM16	* plasmid backbone containing gpdA(p)::neo::trpC(t)*	J. Menzner, KIT
pNH20	* plasmid backbone containing tubA(p); trpC(p)::hph::trpC(t)*	N. Wernet, KIT

**Table 3:** Oligonucleotides used in this study

Name	Sequence (from 5′ to 3′)	Description
pro22_ko_LB_fwd	GATGGCTCGAGTTTTTCAGCAAGATCGATGCCTAACTGATATGGTAG	*ΔsipC*
pro22_ko_LB_rev	GTTGACCTCCACTAGCATTACACTTTTTGATGGTATATTTGGGAGGTG	*ΔsipC*
pro22_ko_hph_fwd	ACCACCTCCCAAATATACCATCAAAAAGTGTAATGCTAGTGGAGGTC	*ΔsipC*
pro22_kp_hph_rev	GGGTCGCCACCTTCTGGCCCTCGTTTGGGGGGAGTTTAGGGAAAG	*ΔsipC*
pro22_ko_RB_fwd	ATGCTCTTTCCCTAAACTCCCCCCAAACGAGGGCCAGAAGGTGG	*ΔsipC*
pro22_ko_RB_rev	ATTGTAGGAGATCTTCTAGAAAGATCCAGTTTGTCAGGTGCTAAAC	*ΔsipC*
pro22_rek_prom_fwd	GATGGCTCGAGTTTTTCAGCAAGATCGAAACTTCTCTGGAAGCGA	*ΔsipC* complementation
pro22_term_rev	GTTGACCTCCACTAGCATTACACTTCCAGTTTGTCAGGTGCTAAAC	*ΔsipC* complementation
pro22_g418_fwd	GTCGGTTTAGCACCTGACAAACTGGAAGTGTAATGCTAGTGGAGGT	*ΔsipC* complementation
pro22_g418_rev	ATTGTAGGAGATCTTCTAGAAAGATTGGGGGGAGTTTAGGGAAA	*ΔsipC* complementation
tubP_hphol_fwd	TGCTCTTTCCCTAAACTCCCCCCAGTAGCTGCCCAGCAAACTC	mCherry-TubA
tubP_RFPol_rev	TACTTACCTCGCCCTTGCTTACCATGATGAATTATATTTCGTCAAGAAGA	mCherry-TubA
RFP_tubPol_fwd	TCTTCTTGACGAAATATAATTCATCATGGTAAGCAAGGGCGAGG	mCherry-TubA
RFP_tubAol_rev	CGTTGATAGAAACAACTTCACGCATTTTGTAGAGTTCATCCATTCCAC	mCherry-TubA
TubA_RFPol_fwd	CGGTGGAATGGATGAACTCTACAAAATGCGTGAAGTTGTTTCTATCAAC	mCherry-TubA
Df_tubA_term_rev	ATTGTAGGAGATCTTCTAGAAAGATATACCGTGTGGCTGCCGAA	mCherry-TubA
lifeact_botgfp_fwd	ATGGGCGTGGCCGACCTGATCAAGAAGTTCGAGAGCATCAGCAAGGAAGAGATGGTTTCCAAGGGTGAGG	Lifeact-GFP
df_tub_rev	TGCAATTGTTGATGTTCAGGC	Lifeact-GFP
tgluc_fwd	CGTATGTAGATAAGATGTATGATTAG	Lifeact-GFP
lifeact_tubOL_fwd	GAAAGCCTGAACATCAACAATTGCAATGGGCGTGGCCGACCT	Lifeact-GFP
gfp_cterm_tglucol_rev	AATCATACATCTTATCTACATACGTTATTTGTAAAGTTCATCCATTCCCA	Lifeact-GFP
p12_lb_hph_ol_fwd	ATGCTCTTTCCCTAAACTCCCCCCAGAGCAGCTCCGAAAGCAAAG	P12-mCherry
p12_ns_mch_ol_rev	TACTTACCTCGCCCTTGCTTACCATAGTGTCGCAAGTGTAGCCG	P12-mCherry

**Table 4:** Comparison of trap compartment length in the *ΔsipC*-deletion strain, WT, and the re-complemented strain. Each value is displayed as mean ± SD

Δ*sipC*	Compartment length (in µm)				Occurrence (in %)
	1	2	3	4	
I	31 ± 3	32 ± 3	35 ± 4	39 ± 5	77 ± 2
II	33 ± 8	34 ± 1	33 ± 3	37 ± 10	13 ± 3
III	28 ± 3	32 ± 11	34 ± 6	48 ± 11	8 ± 1
IV	31 ± 9	33 ± 6	32 ± 12	—	1 ± 1
WT	14 ± 2	17 ± 1	18 ± 1	17 ± 1	—
Re-compl.	19 ± 2	21 ± 1	20 ± 1	19 ± 1	—

To induce trap formation, the fungal strains were cultivated on thin low-nutrient agar (LNA) slides by adding around 1× 10^4^  *D. flagrans* spores and around 100–500 *C. elegans* individuals. Co-incubation was carried out at 28°C in darkness for 12–48 h. *C. elegans* strains were cultivated at 20°C on nematode-growth medium seeded with *Escherichia coli* strain OP50 as food source.

### Targeted gene deletion of *sipC*

*sipC* was deleted by homologous recombination. One kb flanks homologous to the 5’ and 3’ regions of *sipC* were amplified by PCR with primers with 25 bp overhangs homologous to the hygromycin-B resistance cassette as well as to the pJET1.2 plasmid backbone (Thermo Fisher), digested with *Eco*RV (Tables 2 and 3). The hygromycin-B resistance cassette *hph* was amplified using plasmid pFC332 as template, and all fragments were assembled into pJET1.2 using the NEBuilder HiFI DNA Assembly Cloning Kit [New England Biolabs (NEB), Frankfurt]. The fragments were amplified using Q5 polymerase (NEB) and the manufacturer’s recommended protocol. Standard protocols were used for *E. coli* transformation and plasmid isolation. The gene deletion construct was introduced into *D. flagrans* protoplasts as described in Youssar *et al.* (2019). For selection of transformants, PDA plates supplemented with 100 µg/ml hygromycin-B were used. To reintroduce the full-length target gene into the deletion strains, the whole gene including its 1 kb upstream and 0.5 kb downstream regulatory regions was amplified by PCR. To select for positive transformants, the geneticin resistance cassette [*trpC(p)::neo::trpC(t)*] was used. Both fragments were assembled into the pJET1.2 plasmid backbone using the NEBuilder HIFI DNA Assembly Cloning Kit. The complementation plasmid was introduced into the respective mutant strain via protoplast transformation. For selection of transformants, PDA plates supplemented with 150 µg/ml geneticin sulfate (G418) were used.

### Verification of homologous recombination

To extract genomic DNA from *D.* *flagrans*, around 1× 10^8^ spores were inoculated in potato dextrose broth and incubated in a 6-cm diameter Petri dish at 28°C for 48 h. Afterward, the mycelium was collected, and DNA was extracted using the protocol described in Zhou *et al.* (2018). Southern blot analysis was done with digoxigenin (DIG)-labeled probes. Genomic DNA was digested with the appropriate restriction enzyme overnight, separated in a TAE agarose gel and transferred to a nylon membrane. Probes were synthesized using the PCR DIG Probe Synthesis Kit (Roche) according to the manufacturer’s protocol. After probe hybridization, the membrane was hybridized with anti-DIG-AP Fab fragments (Roche) and development was performed using CDP-Star solution (Roche).

### Localization of proteins with GFP and mCherry

####  

***mCherry-TubA***: TubA was tagged with mCherry at the N-terminus expressed from the natural promoter. Using Gibson assembly, PCR-amplified fragments of the hygromycin resistance cassette *hph*, 1 kb *tubA* promoter, *GFP*, and *tubA* open reading frame plus 1 kb downstream regulatory region were cloned into a pJET1.2 plasmid backbone. To express the construct in *D. flagrans* mutant strains, the *tubA(p)::mCherry::tubA::tubA(t)* fragment was cloned into pJM16 containing the G418 resistance cassette. Plasmids and oligonucleotides are listed in Tables 1 and 2.

####  

***Lifeact-GFP***: Lifeact was tagged with GFP at the C-terminus expressed from the *tubA* promoter (Tables 1 and 2). The 51-bp sequence coding for lifeact was included in the forward primer to amplify GFP and subcloned into a pJET1.2 plasmid backbone. Using Gibson assembly, the PCR-amplified Lifeact-GFP fragment was cloned into pNH20 containing the *tubA* promoter and a hygromycin-B resistance cassette. To express the construct in *D. flagrans* mutant strains, the *tubA(p)::lifeact::gfp::gluC(t)* fragment was cloned into pJM16 containing the G418 resistance cassette.

### Transcription reporter assay

To localize the expression of *sipC* in *D. flagrans* during different developmental stages, the *sipC* promoter was fused to the fluorescent reporter gene construct *h2b-mCherry*. The promoter fragment was amplified by PCR and assembled into the plasmid backbone pVW56 containing the *h2b::mCherry* reporter and the *hygromycin-B* resistance cassette by Gibson assembly. The plasmid was transformed into *D. flagrans* sVW10 expressing *h2b(p)::h2b::GFP*.

### Quantification of compartment length, conidiation, and trap morphogenesis

To quantify compartment length of vegetative mycelium, around 1000 spores of the respective *D. flagrans* strain were inoculated on LNA at 28°C for 24 h. The cell wall was stained with Calcofluor White and images were taken at 100× magnification. For each strain, the length of ten compartments of five germinated spores was quantified and performed in three replicates.

To quantify the amount of conidia produced by the different *D. flagrans* strains, 8-mm diameter hyphal discs were punched from the edges of a 7-day-old cultured PDA plate, transferred into 500 µl dH_2_O, and vortexed for 5 min. Conidia were quantified using a Helber counting chamber. Each biological replicate was quantified four times. To quantify chlamydospore quantities and morphology in the Δ*sipC* strain, 1000 spores of wild type (WT), Δ*sipC*, and rescue strain were incubated on LNA at 28°C. After 72 h, chlamydospore number and morphology were assessed. To quantify the morphology of fungal traps, around 1000 spores of the respective *D. flagrans* strains were co-inoculated with around 200 *C. elegans* larvae on thin LNA pads (2× 2 cm) at 28°C for 48 h. The traps were counted and quantified using 100× and 400× magnification. For each strain, three biological replicates were quantified.

### Microscopy

For microscopy, around 1× 10^4^ spores were inoculated on thin LNA (1% agar 1.66 mM MgSO_4_, 5.4 µM ZnSO_4_, 2.6 µM MnSO_4_, 18.5 µM FeCl_3_, 13.4 mM KCl, 0.34 µM biotin, and 0.75 µM thiamin) and incubated for at least 2 h at 28°C. For conventional fluorescence images, a Zeiss AxioImager Z.1 microscope with either Plan-Apochromat 63×/1.4 Oil DIC, EC Plan-Neofluar 40×/0.75, EC Plan-Neofluar 20×/0.50, or EC Plan-Neofluar 10×/0.30 objective and an AxioCamMR was used. The images were taken using ZEN 2012 Blue Edition.

For confocal imaging, a Zeiss LSM900 microscope with Plan Apochromat 63×/1.4 Oil objective and Airyscan 2 detector in super resolution mode was used.

### Fluorescent dyes and inhibitors

To visualize the cell wall of the fungus, the mycelium was stained using Calcofluor-white (CFW, fluorescent brightener 28; Sigma-Aldrich). The 1% (wt/vol) stock solution was prepared by dissolving CFW in 0.5% (wt/vol) KOH and 83% (vol/vol) glycerol. A 1:1000 dilution working solution (in dH_2_O) was added to the mycelium and incubated for 3 min to stain the cell wall before imaging.

A stock solution (1 mg/ml) of the anti-MT agent methyl 1-(butylcarbamoyl)-2-benzimidazolecarbamate (benomyl; Sigma) was prepared in dimethyl sulfoxide (DMSO) and used at a final working concentration of 5 µg/ml in dH_2_O. A stock solution (1 mg/ml) of the F-actin depolymerizing drug Cytochalasin A was prepared in DMSO and used at a final working concentration of 5 µg/ml in dH_2_O. For the inhibitors, a 0.5× 0.5-cm agar pad with mycelium was incubated in 100 µl working solution of each inhibitor and incubated for up to 60 min prior to imaging.

### Statistical analysis

*P*-values were calculated using the mean of three biological replicates and the *t*-test with the software GraphPad Prism 9. In the bar graphs, each biological replicate is color-coded (orange, blue, gray). While each data point is depicted as dot, the averages are shown as triangles (Lord *et al.* 2020).

## Results

### Conservation of the STRIPAK signaling complex in *Duddingtonia flagrans*

**Figure 1:**
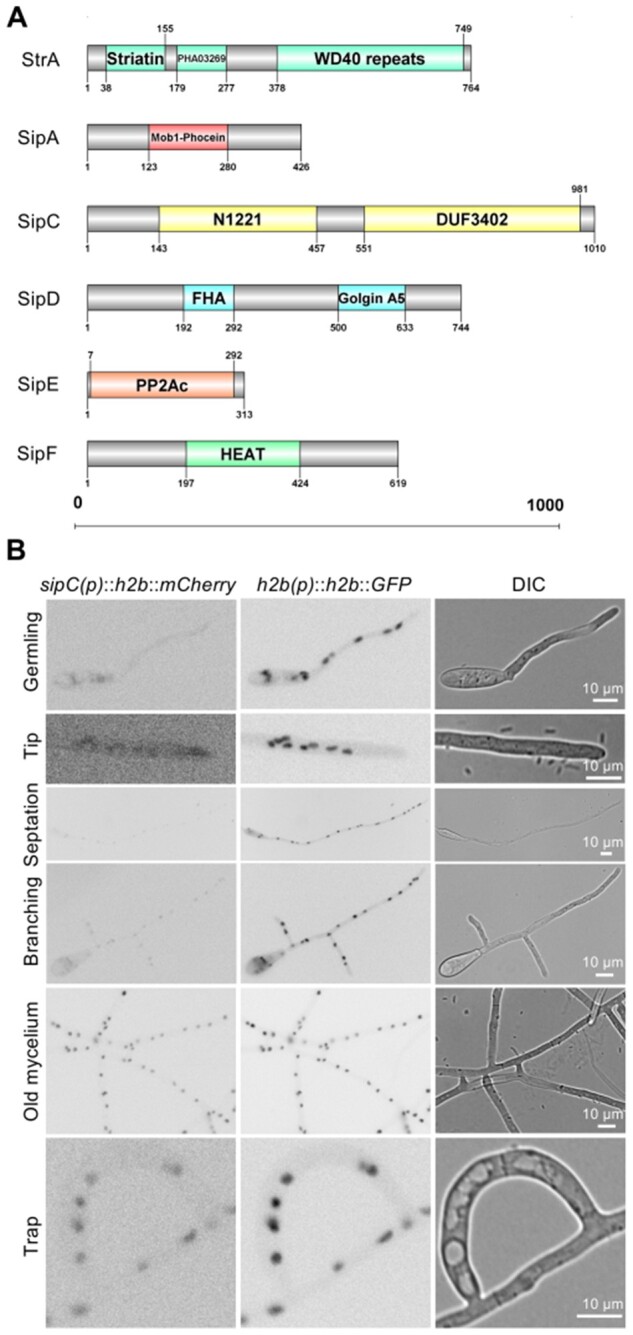
Scheme of *D. flagrans* STRIPAK orthologs and expression of *sipC* in hyphae and traps*.* (A) Domain organization of *D. flagrans* STRIPAK components. The amino acids are numbered, and conserved domains are labeled. Abbreviations: WD40, WD or beta-transducin repeat sequence; FHA, forkhead-associated; HEAT, Huntingtin, elongation factor 3, protein phosphatase 2A, and the yeast kinase TOR1; Mob1, monopolar spindle one-binder protein; N1221, acidic domain with possible transmembrane domains; DUF3402, domain of unknown function 3402; PHAO03269, envelope glycoprotein C. (B) Visualization of the expression of *sipC* using a H2B-mCherry reporter construct. The expression of *h2b-GFP* under the *h2b* promoter was used as a control. Exposure time: mCherry 300 ms, GFP 200 ms. Fungal germlings were observed after 1–2 h of incubation on LNA. Old mycelium refers to at least 16 h postinoculation of spores.

To unravel a role of the STRIPAK complex in trap formation, we identified orthologs of *S. macrospora* PRO11, PP2AA, PP2Ac1, PRO22, SmMOB3, PRO45, SmKIN3, SmKIN24, and SCI1 in *D. flagrans* (Figure 1). They all share conserved domains with homologs from other fungi, suggesting that *D. flagrans* harbors a functional STRIPAK complex. In *A.* *nidulans*, the STRIPAK complex consists of the striatin scaffold protein StrA and six striatin (StrA) interacting proteins (Sip), and the heptameric complex is involved in the regulation of light-dependent development, the production of secondary metabolites, and the stress response (Elramli *et al.* 2019). We followed the nomenclature of *A. nidulans* for the *D. flagrans* STRIPAK proteins and named the proteins *s*triatin StrA and *S*trA-*i*nteracting *p*roteins (SipA-F). StrA [DFL_002622; 764 amino acids (aa)] contains a conserved striatin domain consisting of a caveolin-binding domain and a calmodulin-binding domain. Additionally, it contains WD40 repeats at the C-terminus. SipA (DFL_000993; 426 aa) is an ortholog of Mob3 (Phocein) containing the conserved Mob1-Phocein domain. The ortholog of STRIP1/2 SipC (DFL_007682; 1010 aa) harbors the putative N1221 family domain and a domain of unknown function (DUF3402). The SLMAP protein SipD (DFL_001177; 744 aa) harbors a forkhead-associated (FHA) domain. The ortholog of the catalytic subunit of the phosphatase PP2Ac SipE (DFL_003255; 313 aa) contains the catalytic domain of protein phosphatase 2A. The ortholog of the phosphatase regulatory subunit PP2AA SipF (DFL_008478; 619 aa) contains HEAT repeats (*H*untingtin, *e*longation factor 3, protein phosphatase 2*A*, and the yeast kinase *T*OR1). Two putative STRIPAK-associated GCKs DFL_002427 and DFL_007266 were identified. No ortholog for SipB was found.

**Figure 2:**
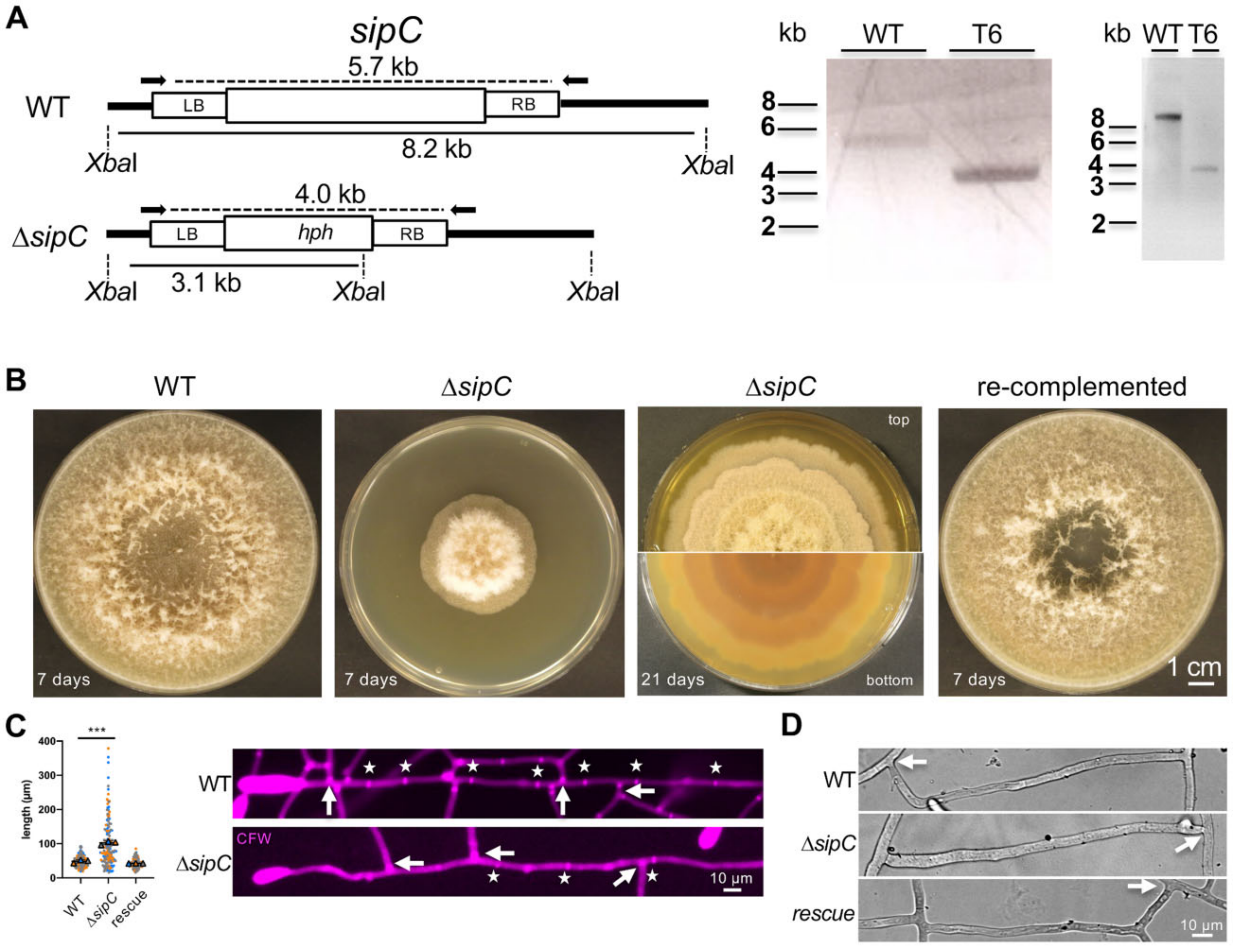
Deletion of *sipC* impairs vegetative growth and septation. (A) Targeted deletion of the *sipC* gene in *D. flagrans* using homologous recombination. Left: scheme of the deletion strategy. Right: analytic PCR using primers indicated on the left (as arrows) and southern blot analysis (outer right panel) using the probe indicated in the scheme. Genomic DNA of wild type (WT) and transformant no. 6 (T6) was digested with *Xba*I before blotting and hybridizing. (B) Growth of *D. flagrans* WT, the *ΔsipC* mutant T6, and the re-complemented strain on PDA after 7 days. Additionally, top and bottom view of the *ΔsipC* mutant on PDA after 21 days are shown. (C) Septation of vegetative mycelium in WT, the *ΔsipC* mutant, and the re-complemented strain. Septa were stained with Calcofluor White. Asterisks indicate septa of the main hypha. Arrows indicate septa at branching hyphae. Quantification of compartment length in each strain. Each biological replicate is color-coded (orange, blue, gray). While each data point is depicted as dot, the averages are shown as triangles. Error bars show the standard deviation (SD). (D) Hyphal fusion events in WT, *ΔsipC*, and the re-complemented strain are labeled with white arrows.

### STRIPAK *sipC* expression

As a first step to characterize the functions of the STRIPAK complex in *D. flagrans*, we analyzed the *sipC* gene. The open reading frame comprises 3528 nucleotides and is disrupted by nine introns [confirmed by RNAseq data (Youssar *et al.* 2019)]. To test if the expression levels change in different hyphae, a microscopic reporter assay was used. To this end, we fused the *sipC-*promoter to the fluorescent reporter gene *mCherry* which was further linked to the histone *h2b* gene (*h2b*), resulting in red fluorescent nuclei when the promoter is active (Yu *et al.* 2021). As a control, the fluorescent reporter gene *h2b-GFP* was expressed with the *h2b*-promoter. The overall expression of *sipC* was weak. No fluorescence was observed in early stages, 4 h after germination of conidiospores, or in the first 10–30 µm of the hyphae (Figure 1B). Fluorescence started to appear in nuclei when the first septum or hyphal branches became visible. Higher levels of expression were observed in older parts of the mycelium (16 h incubation). During trap formation, no significant changes in expression were detected, and the fluorescence of the reporter construct resembled the intensity of later-stage mycelium. These results suggest a function after germ-tube formation during hyphal differentiation.

### *sipC* is required for fast vegetative growth and septation

**Figure 3:**
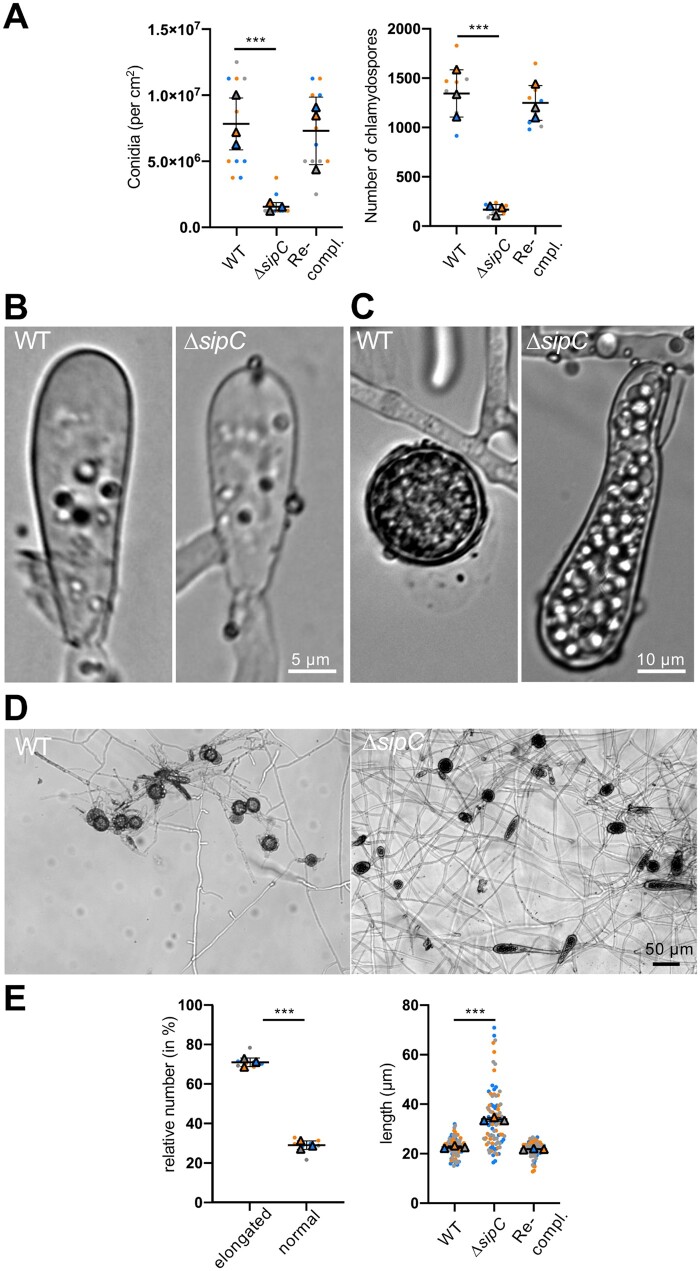
SipC is required for asexual development and determines chlamydospore morphogenesis. (A) Quantification of the number of conidia and chlamydospores in WT, the *ΔsipC* mutant, and the re-complemented strain. (B) Microscopic pictures of conidia of the WT and the Δ*sipC*-mutant strain. (C) Microscopic pictures of chlamydospores of the WT and the *ΔsipC*-mutant strain. (D) Overview of chlamydospores of the WT and the Δ*sipC*-mutant strain. (E) Quantification of chlamydospore morphology in the *ΔsipC* strain and quantification of chlamydospore length of the WT, the *ΔsipC* mutant, and the re-complemented strain. Each biological replicate is color-coded (orange, blue, gray). While each data point is depicted as dot, the averages are shown as triangles. Error bars show the SD.

Since *sipC* was expressed during septum formation, we next generated a *sipC*-deletion strain by replacing the gene with a hygromycin-B resistance cassette by homologous recombination. Twenty-nine hygromycin-B resistant transformants were obtained and screened by PCR and Southern blot analysis. One transformant showed the expected integration and was used for further studies (Figure 3A).

Deletion of *sipC* resulted in slow-growing colonies. While the WT completely covered the medium on a 9-cm Petri dish in 7 days when center inoculated, the *ΔsipC* mutant needed around 21 days (Figure 3B). The formation of aerial mycelium is a common feature of *D. flagrans* on PDA medium. In the *ΔsipC*-deletion strain, it was restricted to the first 1–2 cm of the initial inoculation site. Additionally, the *ΔsipC* mutant showed a 1-cm ring-like growth pattern on PDA. The mutant phenotype could be fully re-complemented with an ectopic copy of the *sipC* gene, including 1 kb upstream and 0.5 kb downstream regulatory elements.

Since we observed the start of expression of *sipC* during septum formation, we quantified the number of septa in the *ΔsipC*-deletion strain after inoculating spores on a low-nutrient medium and incubation for 24 h (Figure 3C). Generally, growth of the *ΔsipC* strain was slower in comparison to the WT and the respective re-complementation strain. While hyphal compartments in WT and the re-complemented mutant strain had a length of 48.5 ± 5 µm [mean standard deviation (SD)] and 42.6 ± 0.3 µm, respectively (*n* = 150), the compartment size was doubled in the* ΔsipC*-deletion strain with a length of 102 ± 5 µm. The differences were still present after an additional 24 h of incubation, resulting in decreased numbers of septa compared to WT. In the *ΔsipC*-deletion strain, septa were mainly found at branching points. Interestingly, whereas the *Δpro22* mutant in *S. macrospora* and *Δham-2* mutant in *Neurospora* *crassa* failed to undergo cell–cell fusion (Xiang *et al.* 2002; Bloemendal *et al.* 2010), in the *D. flagrans* *ΔsipC*-deletion strain, the process appeared unaffected (Figure 3D). The frequency of hyphal fusion in the *ΔsipC*-deletion strain was difficult to compare to WT, since growth speed was heavily reduced.

### SipC is required for asexual development and determines chlamydospore morphology

**Figure 4:**
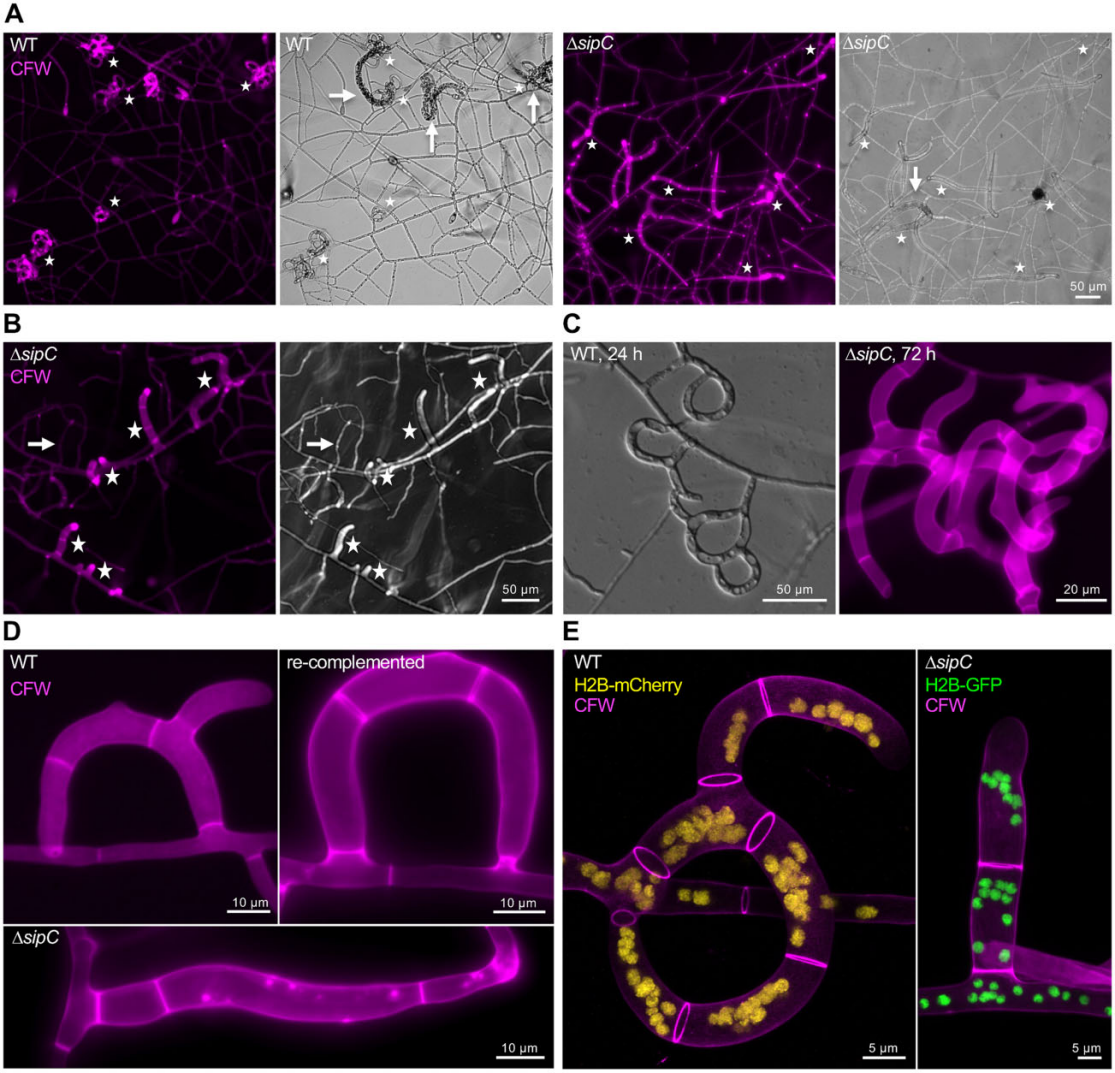
SipC is required for trap formation. (A) Comparison of trap formation in WT, the *ΔsipC* mutant, and the re-complemented strain. The asterisks indicate traps. Trapped *C. elegans* larvae are highlighted by arrows. (B) Overview of the trap formation in the *ΔsipC* strain. The asterisks indicate traps. A vegetative hypha is highlighted by an arrow. (C) Comparison of trapping networks in WT and the *ΔsipC* mutant. (D) Close-up comparison of traps in the WT, the *ΔsipC* mutant, and the re-complemented strain. (E) Visualization of nuclei inside of traps of the WT and the* ΔsipC* mutant. In the WT, the nuclei were labeled by H2B-mCherry (colored in yellow). In the *ΔsipC* mutant, the nuclei were labeled by H2B-GFP (colored in green). The cell wall was stained by CFW (colored in magenta). 3D reconstruction.

Deletion of *sipC* heavily influenced the number of asexual conidia production, resulting in an 80% reduction of conidia of 7-day-old cultures compared to WT (Figure 4A). Likewise, the amount of chlamydospores produced after 72 h on LNA was reduced by 88% compared to WT. In addition, the morphology of chlamydospores was drastically altered, with 71 ± 3% highly elongated and expanded spores in the mutant (*n* = 3) (Figure 4, C–E). All chlamydospores of the WT had a round shape with an average diameter of 22.7 ± 0.5 µm compared to the chlamydospores of the *ΔsipC*-deletion strain which had a length of 33.9 ± 0.7 µm. Yet, these spores were still able to germinate. Chlamydospores develop from differentiated vegetative hyphae, while conidia are formed at the tip of conidiophores. Chlamydospores are formed in older mycelium by condensing the cytoplasm within a compartment into a thick-walled spore. This formation is rather difficult in the *ΔsipC*-deletion strain since the compartment size is much larger than in WT.

### SipC determines the fate of trap cells

**Figure 5:**
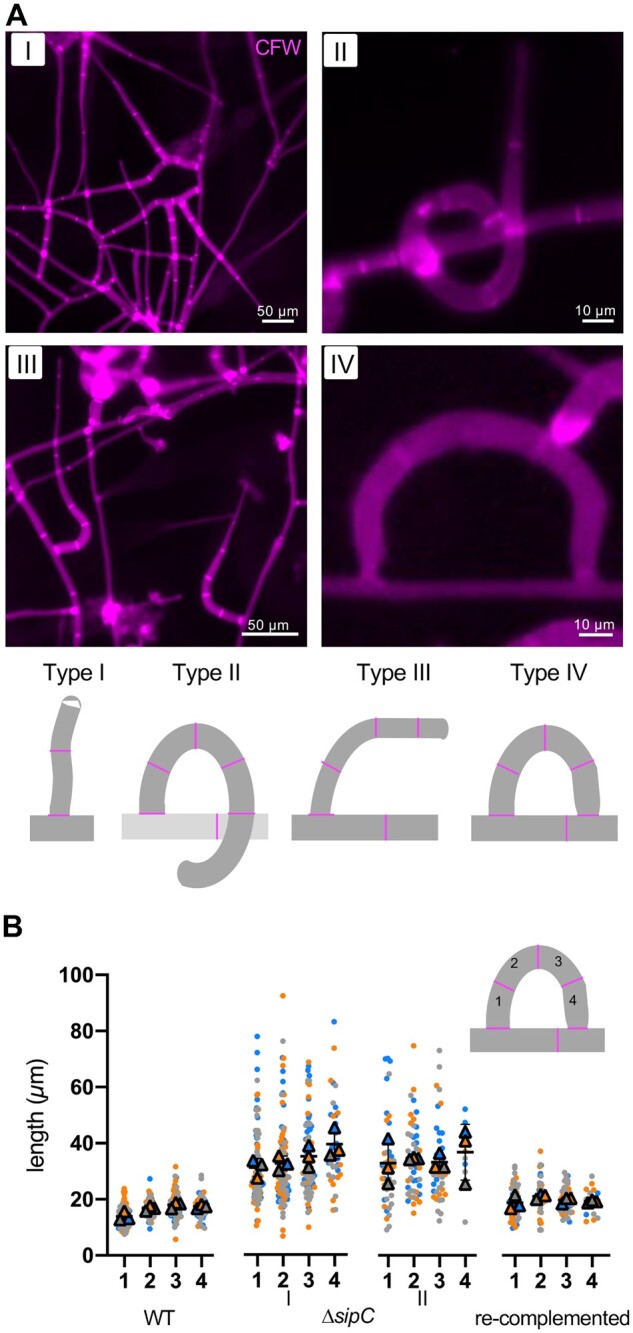
SipA determines trap morphology. (A) Traps of the *ΔsipC* mutant were divided into four groups according to their morphology. I, stick-like trap; II, ring-like trap; III, 90° bending; IV, ring with cell fusion. The cell wall was stained by CFW. (B) Quantification of the compartment length of traps in the WT, the *ΔsipC* mutant, and the re-complemented strain. See Table 4 for the quantification. The length of four compartments was measured. In the *ΔsipC* mutant, quantification of the most abundant groups I (stick-like traps) and II (ring-like traps) are displayed. Each biological replicate is color-coded (orange, blue, gray). While each data point is depicted as dot, the averages are shown as triangles. Error bars show the SD.

To investigate trap morphogenesis of the Δ*sipC*-deletion strain, *C. elegans* was co-incubated with fungal spores on LNA slides for at least 48 h. While trap formation in WT and the re-complemented strain showed ring-like trap networks, this was heavily disturbed in the *ΔsipC*-deletion mutant (Figure 5A). Trap loops were often incomplete and resulted in column-like structures. These column-like traps were either identified and distinguished from normal vegetative hyphae by comparing the hyphal diameter or by an increased fluorescence after CFW staining (Figure 5B). An already formed trap can be a new starting point for another trap resulting in more complex trapping networks. Compared to WT, this phenomenon was restricted in the *Δ*sipC mutant (Figure 5C). Trap morphology was restored in the re-complemented strain (Figure 5D). The number of nuclei in trap cells in WT and the *ΔsipC*-deletion mutant strain showed no apparent difference (Figure 5E).

**Figure 6:**
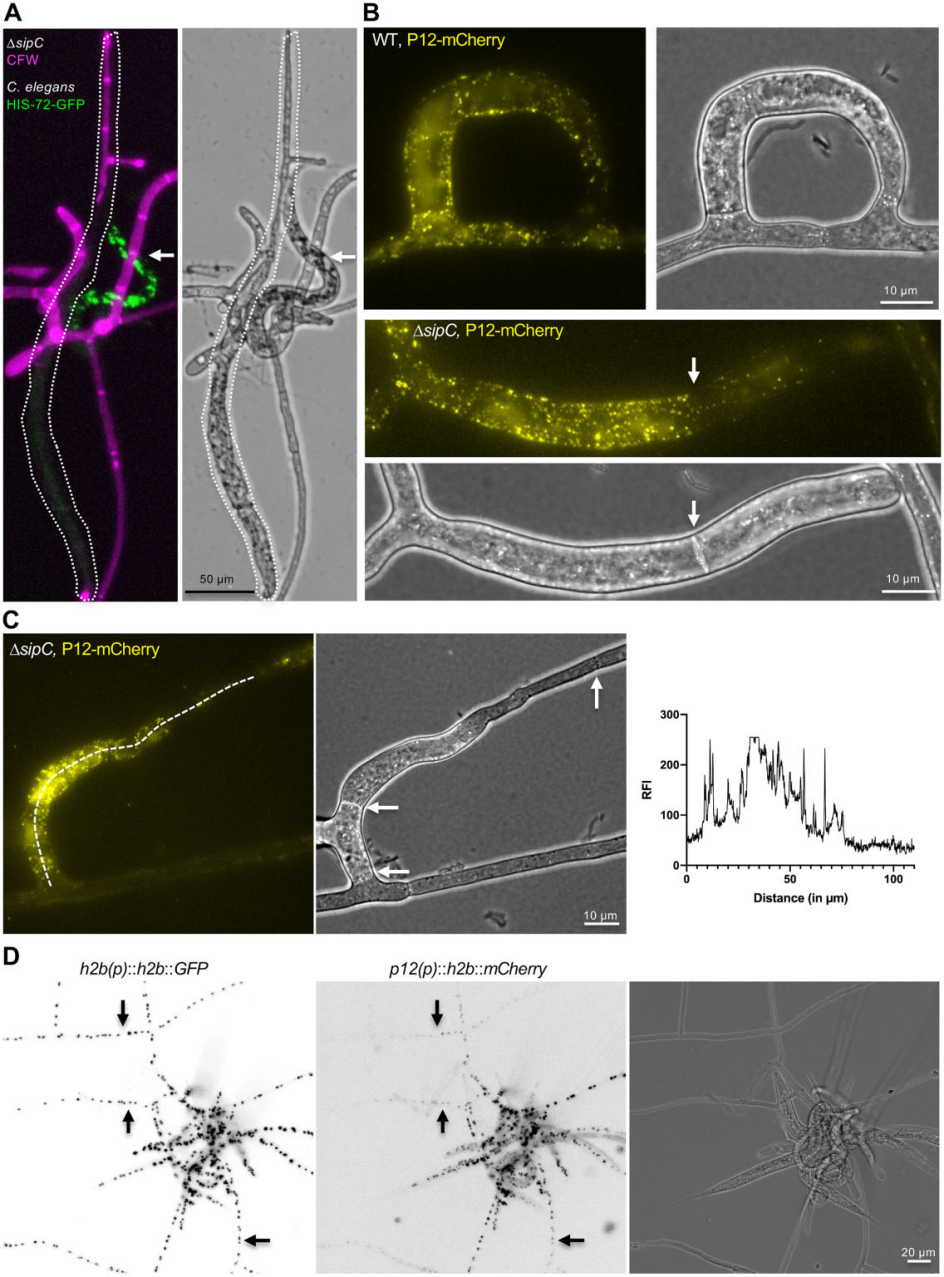
SipA controls the expression of trap-specific genes. (A) Trapping of *C. elegans* by traps of the Δ*sipC* mutant. The cell wall of the fungus is stained by CFW. A *C. elegans* strain expressing a C-terminal GFP fusion protein of the histone HIS-72 was used to distinguish between digested (surrounded by a dashed line) and trapped but alive (arrow) nematodes. (B) C-terminal mCherry tagging of the serine-protease P12 (colored in yellow) in the WT and the *ΔsipC* mutant. The arrow shows a septum and indicates the switch from a trap cell to vegetative growth. (C) The relative fluorescent intensity (RFI) along a *ΔsipC* trap was measured and plotted (*x*-axis displays the distance in µm; *y*-axis displays the measured RFI). Arrows indicate the septa of the trap. (D) Visualization of the expression of *p12* during trapping of *C. elegans* using a H2B-mCherry reporter construct. The expression of H2B-GFP under the *h2b* promoter was used as control. Arrows indicate transition points of reduced *p12*-mCherry expression in vegetative hyphae.

**Figure 7:**
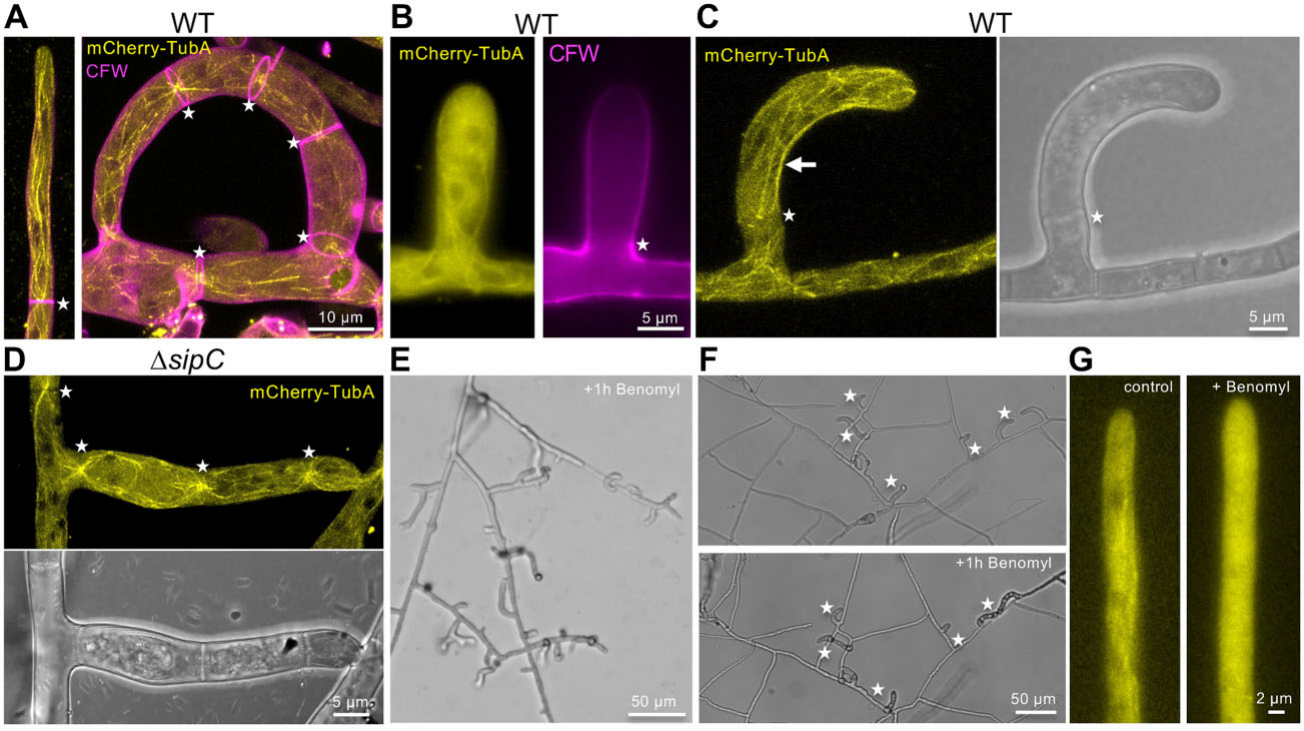
Characterization of the microtubule cytoskeleton. (A) Visualization of an N-terminal mCherry fusion protein of the alpha-tubulin TubA in a vegetative hypha and trap of *D. flagrans* WT (colored in yellow). The cell wall was stained by CFW (colored in magenta). Asterisks indicate the localization of septal MTOCs. Three-dimensional reconstruction. (B) Microtubules in a forming trap, where still no septum was formed at the base (asterisk). (C) MTs in a bending trap. The asterisk indicates a septal MTOC and the presence of a septum. The arrow points to microtubule bending at the inner site of the trap. (D) Localization of mCherry-TubA in a trap of the *ΔsipC* mutant. (E) Hyperbranching of vegetative hyphae after 1 h treatment with the microtubule-depolymerizing drug benomyl (5 µg/ml). (F) The addition of benomyl (5 µg/ml) stopped trap morphogenesis in *D. flagrans* WT. Forming traps are labeled with an asterisk. (G) Disassembly of MTs after 5 min treatment with benomyl (5 µg/ml).

Traps of the *ΔsipC-*deletion strain were divided into four groups according to the morphology of the trap, and the occurrence was quantified (Figure 6A). Of all counted traps, 77 ± 2% showed a column-like structure (I), 13 ± 3% a ring-like structure without cell fusion (II), 8 ± 1% a 90 degrees bending (III), and 1 ± 1% a ring with cell fusion (IV) appearance (total trap number in three biological replicates were *n* = 224, *n* = 156, and *n* = 276, respectively; Figure 6B). In contrast, mostly traps of group II and IV occurred in WT and the re-complemented strain. Despite the morphological differences, traps of the *ΔsipC-*deletion strain were still adhesive, and the strain was able to immobilize and digest nematodes (Figure 7A).

Traps in WT usually consist of two to four compartments before fusing to the neighboring basal hypha. In contrast, compartments of the traps in the *ΔsipC-*deletion strain were highly elongated as in vegetative mycelium. Interestingly, the elongated trap cells often switched to a vegetative growth mode after one, or a maximum of three trap compartments, indicated by a thinner diameter of the hyphae. The change of cell fate from trap cells to normal vegetative hyphae was confirmed by expressing the ortholog of the trap-specific serine protease P12 (DFL_008096) tagged with mCherry in the WT and the *ΔsipC* mutant background. Serine proteases have been identified as crucial virulence factors upon exposure to nematodes, and the *A. oligospora* P12 was highly upregulated during nematode exposure (Yang *et al.* 2011; Zhen *et al.* 2018). P12-mCherry localized in dynamic spots inside the traps of the WT and the *ΔsipC* mutant (Figure 7B). The change of cell fate from trap cells to normal vegetative hyphae in traps of the *ΔsipC*-deletion strain was indicated by the absence of fluorescent accumulations during trap morphogenesis. This change also coincided with a change in hyphal diameter (Figure 7C). Additionally, we observed dynamic spots of P12-mCherry with weaker intensities in neighboring compartments. Therefore, we visualized the promoter activity of *p12* as described above by fusing the *p12* promoter to the fluorescent reporter construct of H2B-mCherry and transformed the plasmid into a strain expressing H2B-GFP under the control of the *h2b* promoter. Promoter activity was also present in neighboring cells but decreased in more distant vegetative hyphae (Figure 7D).

Since the morphology of the traps was drastically changed in the *ΔsipC-*deletion strain, we hypothesized that the deletion of *sipC* influences the dynamics of the cytoskeleton. The cytoskeleton has never been visualized in living traps of NTF. Therefore, we visualized MTs and F-actin by tagging the *D. flagrans* alpha-tubulin TubA with mCherry and the actin-binding peptide LifeAct with GFP. In filamentous fungi, the minus ends of MTs locate at spindle pole bodies which serve as MT-organizing centers (MTOC). Additionally, areas close to the septum can act as septal MTOC (sMTOC) (Zekert *et al.* 2010; Gao *et al.* 2019). In the tip compartments, we observed dynamic MTs reaching the hyphal tip (Figure 8A). In trap cells, the most visible MTs nucleated from septal MTOCs (Figure 8A). Interestingly, major bending of the trap cell was only visible after the first septum at the base (Figure 8, B and C). Although MTs were dynamic, long MTs were observed at the inner side of the trap, reaching from the septum to the tip. In the* ΔsipC* mutant, MT localization appeared similar (Figure 8D). To study the role of MTs during trap morphogenesis, we added cytoskeleton-destabilizing drugs during trap development. The presence of benomyl (5 µg/ml) led to hyperbranching of the mycelium (Figure 8E). Hyphal growth during trap development stopped after the addition of benomyl (Figure 8F). Depolymerization of MTs was confirmed by imaging mCherry-labeled MTs after the addition of benomyl (Figure 8G).

**Figure 8:**
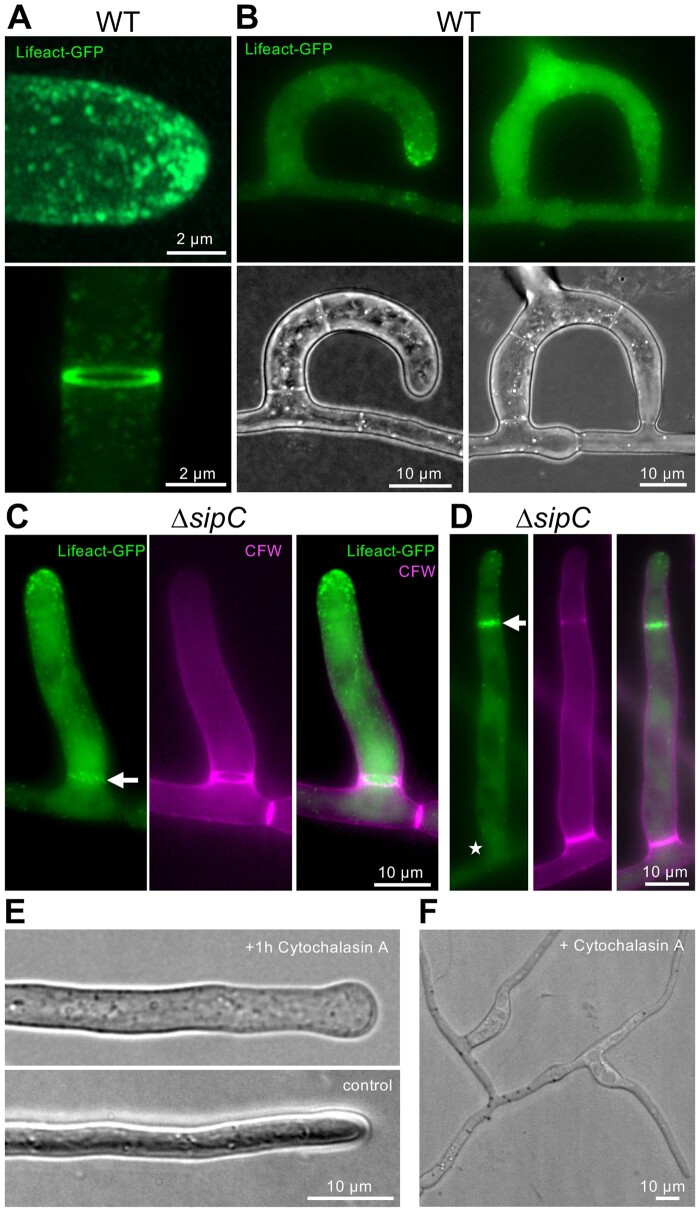
Characterization of the actin cytoskeleton. (A) Actin patches at a hyphal tip and an actin-ring during septum development visualized with Lifeact-GFP (colored in green). Three-dimensional reconstruction. (B) Actin during trap formation and in a mature trap. (C) Actin trap morphogenesis in the *ΔsipC* mutant strain. The white arrow indicates the septum at the base. The cell wall was stained by CFW. (D) Septum formation in a column-like trap in the Δ*sipC* mutant strain. The arrow indicates septum development by localization of Lifeact-GFP at a developing septum. The asterisk indicates a mature septum without GFP signal. (E) The addition of the actin-depolymerizing drug cytochalasin A (5 µg/ml) leads to swollen hyphal tips. As solvent control, hyphae were treated with 0.5% DMSO. (F) Loss of trap identity after the addition of cytochalasin A (5 µg/ml). After some time, traps continued to grow as vegetative hyphae (see also Supplementary Movie S1).

Next, we visualized actin using the actin-binding peptide LifeAct-GFP (Riedl *et al.* 2008). Mostly, actin patches at the hyphal tip and subapical regions as well as the actomyosin ring during septum formation were visualized (Figure 9, A and B). Rarely, dynamic actin cables at the apex of hyphae were observed. Expression of Lifeact-GFP in the *ΔsipC* mutant revealed that septum formation was delayed during trap morphogenesis, resulting in elongated cells with decreasing hyphal diameter (Figure 9, C and D). The *ΔsipC*-deletion strain did not show any difference in the sensitivity toward cytochalasin A as compared to WT (data not shown).

**Figure 8:**
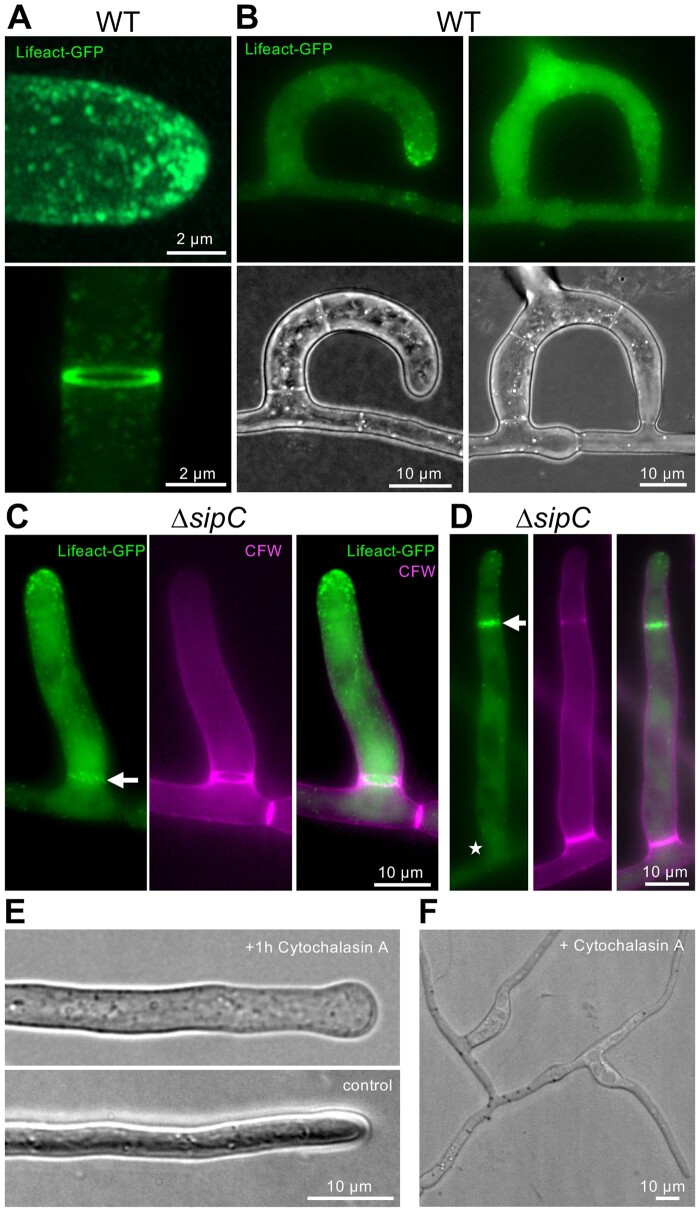
Characterization of the actin cytoskeleton. (A) Actin patches at a hyphal tip and an actin-ring during septum development visualized with Lifeact-GFP (colored in green). Three-dimensional reconstruction. (B) Actin during trap formation and in a mature trap. (C) Actin trap morphogenesis in the *ΔsipC* mutant strain. The white arrow indicates the septum at the base. The cell wall was stained by CFW. (D) Septum formation in a column-like trap in the Δ*sipC* mutant strain. The arrow indicates septum development by localization of Lifeact-GFP at a developing septum. The asterisk indicates a mature septum without GFP signal. (E) The addition of the actin-depolymerizing drug cytochalasin A (5 µg/ml) leads to swollen hyphal tips. As solvent control, hyphae were treated with 0.5% DMSO. (F) Loss of trap identity after the addition of cytochalasin A (5 µg/ml). After some time, traps continued to grow as vegetative hyphae (see also Supplementary Movie S1).

To study the role of actin during trap morphogenesis, we added the F-actin-destabilizing drug cytochalasin A (5 µg/ml) during trap development, which led to swelling of hyphal tips of vegetative mycelium (Figure 9D). This effect was less obvious in growing traps; however, they continued to grow as vegetative hyphae at the end of the reaction (Figure 9E; Supplementary Movie S1). These results suggest a putative function of SipC in regulating cytoskeleton dynamics. However, we did not see apparent differences in the actin organization in the *ΔsipC*-deletion strain as compared to WT.

## Discussion

The STRIPAK complex is involved in many cellular processes in eukaryotes. In filamentous fungi, it is important for hyphal fusion, sexual development, and hyphal growth (Kück *et al.* 2019). In this study, we show that the *D. flagrans* STRIP1/2 ortholog SipC plays a role in septation, conidiation, and trap morphogenesis and is important for cell-fate determination. Although the early steps of trap formation appeared unaffected in a *sipC*-mutant strain, trap cells apparently lost their memory and re-differentiated into normal vegetative hyphae. The same phenotype was observed after disturbance of the actin cytoskeleton. Disturbance of the MT cytoskeleton had a completely different effect and blocked further growth of traps. There are numerous examples for the role of the STRIPAK complex in cytoskeletal functions (Sakuma *et al.* 2015). It was reported that the Strip-dependent regulation of the GCKs MST3 and MST4 modulates actomyosin contraction and therefore cell motility in cancer cells (Madsen *et al.* 2015). Our results raise the question of possible links among the STRIPAK signaling complex, the actin cytoskeleton, and cell-fate determination in the traps of *D. flagrans.*

In filamentous fungi, F-actin forms actin cables, rings, and patches (Berepiki *et al.* 2011). Actin cables are nucleated by formins and localize at the apex of hyphae, where they are involved in polar growth (Riquelme *et al.* 2018). Filamentous fungi usually only encode one formin and deletion of the gene in *A. nidulans* or *N. crassa* is lethal (Sharpless and Harris 2002; Justa-Schuch *et al.* 2010). Interestingly, the STRIPAK subunit PRO45 was found as an interaction partner of formin in *S. macrospora*, although the exact molecular function of the interaction has not yet been solved (Stein *et al.* 2020). It is conceivable that PRO45 influences the activity of formin. Additionally, a class II myosin was found as an interaction partner of the STRIPAK complex component PRO45 in *S. macrospora* (Nordzieke *et al.* 2015). The fact that *D. flagrans sipC* mutants grow very slow may reflect the role of SipC on the actin cytoskeleton in the apical dome. The effect is clearly different from the effect of cytochalasin, which destroys the actin cytoskeleton. In addition to an effect on polar growth, *sipC* mutation had a strong effect on septation. Comparable to the effect on hyphal growth, septation is still possible without SipC, but the timing appears to be different. Although this has no obvious effect on vegetative hyphae, the process appears to be crucial for trap-cell memory. A similar effect was observed during sexual development of the *S. macrospora* mutant Δ*pro22*, where aseptated ascogonia showed a developmental arrest at the protoperithecial stage (Bloemendal *et al.* 2010). This defect resulted in elongated ascogonial coils.

In addition, to a direct effect of SipC on the actomyosin system, there is good evidence in *S. pombe* that the SIN can be regulated by the STRIPAK complex. It was shown that SIP-mediated dephosphorylation of the Ste20 kinase Cdc7p initiates SIN assembly (Singh *et al.* 2011). Misregulation of SIP inhibits the assembly of SIN. In *A.* *nidulans*, the Cdc7p ortholog SepH is required for initiation of septation prior to actin ring assembly. While downregulation of *sepH* results in a loss of septation, overexpression results in hyper-septation (Bruno *et al.* 2001). A similar phenotype was observed in *S. macrospora* in a *Smkin3* mutant. *Smkin3* is the ortholog *of A. nidulans* SepL, and the downstream target of SepH (Radchenko *et al.* 2018). In this study, the deletion of the STRIPAK-complex component *sipC* resulted in a drastic reduction of septa and trap compartments of very different sizes.

Taken together, there are multiple links how SipC may affect the actin cytoskeleton and septation. Compartment size appears to be very important for cell-fate determination during trap formation.

## Data availability

Strains and plasmids are available upon request. The authors affirm that all data necessary for confirming the conclusions of the article are present within the article, figures, and tables. Supplementary material is available at figshare: https://doi.org/10.25386/genetics.16617721.
